# Conflicting Biomedical Assumptions for Mathematical Modeling: The Case of Cancer Metastasis

**DOI:** 10.1371/journal.pcbi.1002132

**Published:** 2011-10-06

**Authors:** Anna Divoli, Eneida A. Mendonça, James A. Evans, Andrey Rzhetsky

**Affiliations:** 1 Department of Medicine, University of Chicago, Chicago, Illinois, United States of America; 2 Institute for Genomics and Systems Biology, University of Chicago, Chicago, Illinois, United States of America; 3 Department of Pediatrics, University of Chicago, Chicago, Illinois, United States of America; 4 Computation Institute, University of Chicago, Chicago, Illinois, United States of America; 5 Department of Sociology, University of Chicago, Chicago, Illinois, United States of America; 6 Department of Human Genetics, University of Chicago, Chicago, Illinois, United States of America; University of Colorado School of Medicine, United States of America

## Abstract

Computational models in biomedicine rely on biological and clinical assumptions. The selection of these assumptions contributes substantially to modeling success or failure. Assumptions used by experts at the cutting edge of research, however, are rarely explicitly described in scientific publications. One can directly collect and assess some of these assumptions through interviews and surveys. Here we investigate diversity in expert views about a complex biological phenomenon, the process of cancer metastasis. We harvested individual viewpoints from 28 experts in clinical and molecular aspects of cancer metastasis and summarized them computationally. While experts predominantly agreed on the definition of individual steps involved in metastasis, no two expert scenarios for metastasis were identical. We computed the probability that any two experts would disagree on *k* or fewer metastatic stages and found that any two randomly selected experts are likely to disagree about several assumptions. Considering the probability that two or more of these experts review an article or a proposal about metastatic cascades, the probability that they will disagree with elements of a proposed model approaches 1. This diversity of conceptions has clear consequences for advance and deadlock in the field. We suggest that strong, incompatible views are common in biomedicine but largely invisible to biomedical experts themselves. We built a formal Markov model of metastasis to encapsulate expert convergence and divergence regarding the entire sequence of metastatic stages. This model revealed stages of greatest disagreement, including the points at which cancer enters and leaves the bloodstream. The model provides a formal probabilistic hypothesis against which researchers can evaluate data on the process of metastasis. This would enable subsequent improvement of the model through Bayesian probabilistic update. Practically, we propose that model assumptions and hunches be harvested systematically and made available for modelers and scientists.

## Author Summary

Mathematical models and scientific theories fail not only from internal inconsistency, but also from the poor selection of basic assumptions. Assumptions in computational models of biomedicine are typically provided by scientists who interact directly with empirical data. If we seek to model the dynamics of cancer metastasis and ask experts regarding valid assumptions, how widely will they agree and on which assumptions? To answer this question, we queried 28 faculty-level experts about the progression of metastasis. We demonstrate an unexpected diversity of assumptions across experts leading to a striking lack of agreement over the basic stages and sequence of metastasis. We suggest a formal model and framework that builds on this diversity and enables researchers to evaluate divergent hypotheses about metastasis with experimental data. We conclude that modeling biomedical processes could be substantially improved by harvesting scientific assumptions and exposing them for formalization and experiment.

## Introduction

When designing a mathematical model about a biological phenomenon, the computational biologist typically follows several paths in search of existing knowledge. She may consult textbooks, scan for relevant research articles, evaluate the prior modeling literature, seek advice from or collaborate with domain experts. In most cases, the pool of all relevant publications is too vast to read, leading to an idiosyncratic sample of viewpoints. Seeking advice from expert colleagues, however, raises the same concern–the unknown degree to which their views represent the distribution of expert opinion across the field. Proper evaluation of the uncertainty about domain knowledge has profound empirical importance for scientific advance and model design [1], [2]. In the short run, improper selection of domain knowledge would make it difficult for a scientist to convince reviewers and readers about the validity of her approach. In the long run, a model based on unpopular or dubious assumptions is less likely to provide a useful predictive description regarding the phenomenon of interest.

Imagine that a model-designer has time and energy to seek advice from a broad sample of colleagues. How coherent is the entire collection expert opinion? Would disagreement be reserved for minor issues and consensus obtain for major ones? Would only low-certainty issues be disputed, or would experts express certainty about opposing answers to central questions? Finally, how can one reliably measure these quantities? While answers to these questions likely vary across domains, few studies in biomedicine or other areas of science have addressed them [3]–[6].

A formal way to think about expert opinion is through the lens of Bayesian statistics [7]. In comparing multiple mathematical models of the same process, we can estimate the posterior probability of a model given data. The model with the highest posterior probability, given the same data, could be said to “describe” the data best. To compute the probability of a model given data, we must specify prior distributions over models and over model-specific parameters. Expert opinion is precisely what should be the basis of these priors. If the amount of experimental data is modest, strong prior distributions over parameter values can profoundly affect the results of statistical analyses. Even more important are prior distributions over the much larger space of models. The results of analysis can be very different under distinct models, and expert confidence in particular models dramatically influences the interpretation of data.

Here we consider a somewhat neglected aspect of biomedical modeling: scientific uncertainty about biological models. This uncertainty only partially transpires in research publications. It predominantly resides in experts' minds and close conversations. We note that in some fields, such as engineering, it is more common to acquire expert opinion and employ probabilistic models when limited information is available [8]. Even there, however, the focus is on ascertaining modal opinion and not estimating diversity and uncertainty. As a case study, we chose the field of cancer metastasis: the spread of cancer cells from their original location to new sites in the body.

Metastasis has enormous medical importance. In 2000 Hanahan and Weinberg published a classic review article entitled “The Hallmarks of Cancer” [9] in which they famously diagrammed six functional capabilities that all cancers seem to share: (1) limitless replicative potential, (2) sustained angiogenesis, (3) evading apoptosis, (4) self-sufficiency in growth signals, (5) insensitivity to anti-growth signals, (6) tissue invasion and metastasis. Among these six hallmarks, metastasis plays the most prominent role in determining a patient's fate. If every malignancy remained at its initial location, most solid cancers could be treated effectively with surgery. Metastasis or the spread of cancer to multiple new locations, however, makes it increasingly hard to track and remove new colonies of afflicted cells. Eventually, the infestation becomes so severe that nothing can be done for the patient beyond reducing pain and administering toxic drugs to slow invasion. Developing high-fidelity mathematical models of the multistage progression of metastasis will be critical for finding new ways to slow or stop it—for example, by identifying vulnerable steps in the process that could be targeted by medicine.

The field of metastasis research is established but very dynamic—over 80% of the literature on metastasis was published during the last two decades and almost 60% in the last ten years—making it an attractive subject for the design of computational models and the assessment of (living) expert opinion. A field in which knowledge claims are more established and stable would admittedly be easier and more reliable to model, and yet such models would unlikely contribute to scientific advance. Metastasis is a complex phenomenon involving a wide community of active researchers with diverse training. This necessarily increases the diversity of opinion within the field, but creates opportunities for modeling that contribute to advance by analytically juxtaposing the consequences of different perspectives. We assessed prior knowledge about metastasis by collecting and analyzing the views of 28 experts. Our analysis has broad implications for modeling biological and scientific phenomena in general, for exposing scientific assumptions for evaluation and testing, for understanding the complex process of peer-review, and for bridging disconnected subfields of biomedicine.

### Stages of metastasis and hypotheses

**Figure 1:**
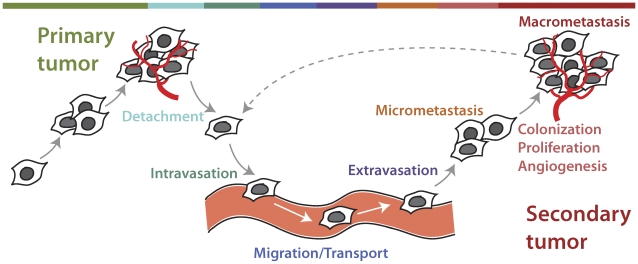
A “textbook” view of the metastatic progression of a malignant tumor. The tumor's development starts with its growth at the primary location (*primary tumor*). In metastatic progression, some cells from the primary tumor detach from the colony (*detachment*), enter blood or lymph vessels (*intravasation*) and travel within the body (*migration/transport*). Next, the traveling cells exit blood or lymph vessels (*extravasation*) and colonize new sites in the body. There, they divide and form tiny colonies at first (*micrometastasis*), followed by further cell proliferation, recruitment of blood vessels (*angiogenesis*) that provide small colonies with sufficient nutrients to develop into large tumors (*macrometastasis*). It is currently unclear if secondary colonies can re-metastasize to form tertiary and quaternary colonies (dotted line indicating a cyclic process).

The accepted (“textbook”) view of metastasis includes the following stages (see Figure 1). At the *primary* stage, an individual renegade cell becomes malignant, begins uncontrollable proliferation, learns to avoid the immune system and anti-growth signals, and forms the initial (primary) tumor with access to its own blood supply. This stage is followed by a stage of *detachment* (migration), where tumor cells disconnect from the primary colony to begin their journey through the body. The next stage involves *invasion*, *breach of the extracellular membrane (ECM)*, and *intravasation*. At this stage the cancer cell has to overcome several obstacles to actively reach and penetrate the wall of a blood or lymph vessel (intravasation), gaining access to the body's transport system that would carry it to unaffected tissues. Next, blood or lymph carries the cancer cell to new locations—the stage of *migration* (transport). The *extravasation* stage is intravasation in reverse: the cancer cell escapes the blood or lymph vessel that has carried it by penetrating its wall and invades the new organ or tissue. This stage is followed by *colonization* of the tissue/organ and cell *proliferation*. Typically, early stages of this infestation are described as *micrometastasis*, formation of a small secondary tumor, usually without its own blood supply (no angiogenesis occurs at early stages) and with a balance of proliferation and apoptosis. When the secondary tumor manages to activate growth of its own blood vessels, it enters the stage of *macrometastasis*, culminating in the growth of a large secondary tumor.

While there is evidence that metastasis was recognized as early as in 250 BCE in China, the term was coined in 1829 by French gynecologist Joseph Recamier [10]. Since that time there have been several important theories about metastasis and its role in the progression of cancer. Some theories attempt to explain how cancer cells gain metastatic potential—how they gain the ability to detach from the base membrane, lose gap and tight junction contacts with neighboring cells, migrate from primary tumor sites, enter and survive in the vasculature or lymphatics before arrest in the secondary site and proliferate within the vessel or, after extravasation, into the surrounding tissue. The “fusion model” explains these abilities by suggesting that epithelial cells from solid tumors somehow fuse with cells from lymphoid tissue (e.g., leukocytes) that possess many of these abilities or that tumor DNA is taken up in the broader circulatory system. This follows from a 19^th^ Century finding that eggs experimentally fertilized with multiple sperm underwent abnormal mitosis, which suggested similar chromosomal imbalance might result in oncogenesis [11]. Alternately, Nowell proposed the “progression model” in 1976 that postulated a series of somatic mutational events required for a cell to acquire a more embryonic phenotype that endowed it with metastatic abilities [12]. As a result, only a small fraction of cells stochastically acquire this ability. Other models suggest a broad genetic predisposition that interacts with either a progression or fusion mechanism [13]. The issue of when and how cancer cells gain metastatic potential is one of the pending questions we examine here.

Another active area of theory development involves the relationship between primary and secondary tumor sites. Perhaps the most famous is the “seed and soil” hypothesis, formulated by Paget [14] in 1889 and based on the following metaphor: “When a plant goes to seed, its seeds are carried in all directions… But they can only live and grow if they fall on congenial soil” [14]. If cancer cells are carried passively from the original tumor to secondary locations, can they colonize any tissue they might encounter? Paget analyzed more than seven hundred case histories of breast cancer and found that metastases formed in the liver far more often than in the spleen, but the spleen had nearly identical exposure to cancer cells as the liver via blood flow. Therefore, the liver must provide more “congenial soil” for breast cancer “seeds” than the spleen. This theory and others were eclipsed in the genetics revolution beginning in the 1950s, when the dominant paradigm in cancer research turned to investigation of “oncogenes” that controlled cell growth. In the last decade, however, many empirical demonstrations have convinced researchers of the importance of Paget's “soil”, now redefined in molecular terms as a tumor context or microenvironment required for secondary tumor growth. The issue of secondary site selection, also known as *tropism*, is another question we examine here.

We also explore expert views on the factors that affect other parts of metastasis, from the entry of cancer cells into the blood stream (intravasation) to the initial growth of secondary tumors (micrometastasis) to the recruitment of blood vessels by those tumors (angiogenesis). We believe that understanding the distribution of expert intuitions on these issues will expose their assumptions to scrutiny and speed the creation of reliable formal models.

## Materials and Methods

### Interviews

We conducted individual interviews with 28 principal investigators studying clinical and molecular aspects of metastasis. Subjects were selected based on their expertise, professional stature, and availability for interview. The interviews took place between March 2008 and April 2009. Experts were recruited through personal contacts and by asking interviewed experts to recommend others at the close of our interview. Experts were specialists in a number of different disciplines: breast, head and neck, prostate, gastrointestinal, urinary, ovarian, and brain cancer; melanoma, neuroblastoma, developmental biology, radiation oncology, immunology, endocrine pathology, genetics, biochemistry, and the cell and molecular biology of cancer. They were located in 10 institutions in four countries (USA, UK, Sweden and Australia). All interviews with experts from the University of Chicago were conducted in person and all others by telephone.

Prior to the interviews, we conducted four informal conversations with four experts: one MD/PhD and three PhD scientists. These exploratory pre-interviews were used to guide design of the study, allowing us to explore and define topics of biological and clinical interest. We linked these topics with description in scholarly articles and used them as a basis for the remainder of the interviews.

**Figure 2:**
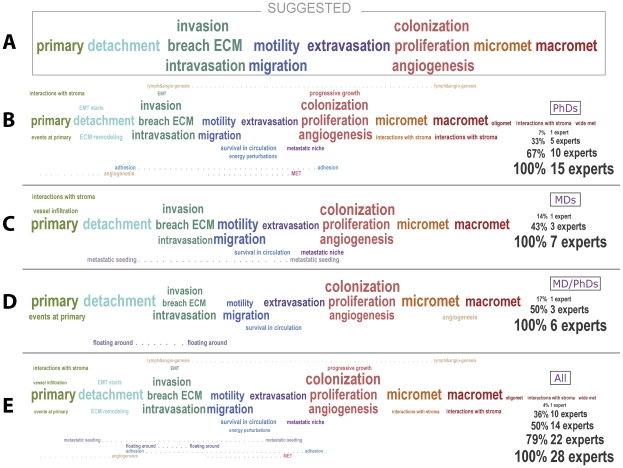
Visualization of expert views about the importance of canonical metastatic stages within the actual process of metastasis. While commenting on the suggested diagram (see Figures 1 and 2A), most experts were not confident that certain stages proposed are part of the metastatic process observed in the laboratory or clinic. Font size represents the number of experts voting for inclusion of the stage represented by the corresponding phrase. (A) The schematic that we presented to experts as canonical. (B–D) Subgroups of experts: PhDs only (B), MDs only (C), MD/PhDs (D). (E) The distribution of certainty across all experts.

**Figure 3:**
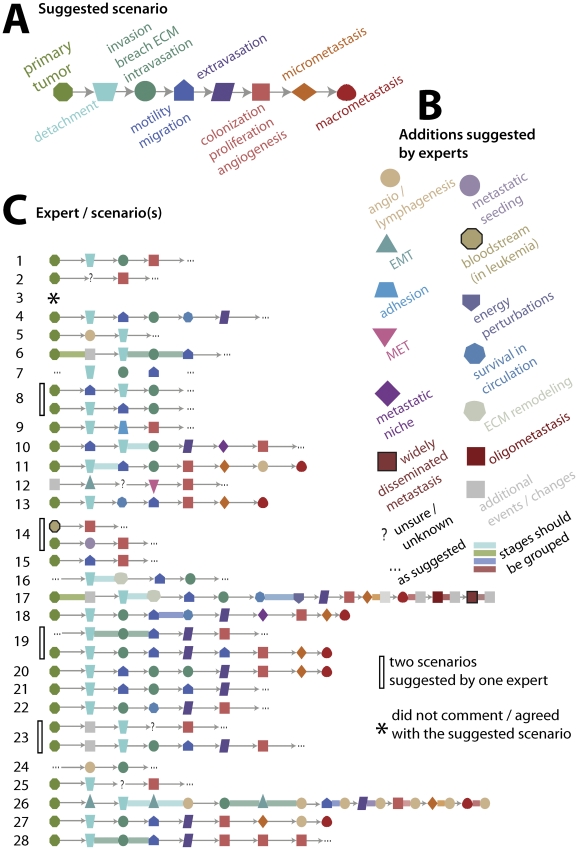
Metastatic cascades as viewed by individual experts. (A) The cascade that we presented to the experts as a “textbook” cascade. Experts suggested reordering stages, removing certain stages and/or adding new ones. (B) Expert-specific depiction of metastasis progression suggested during interviews. Note that every scenario is distinct. Expert 3 did not suggest any scenario, commenting that we have insufficient knowledge; four experts suggested two possibilities (depicted as a pair of scenarios grouped by vertical bars). The ellipses (…) indicate that experts agreed with the prior or posterior sequence that we showed them. (Supplementary Figure S1 demonstrates further variation provided by experts in renaming stages.) (C) Additional stages/concepts that were not present in the original schematic (A) but were suggested by experts.

Our semi-structured interviews had two parts. At the beginning of each, we instructed interviewees to imagine an informal setting in which they could comfortably share speculations and hunches. We then asked experts to provide a definition of metastasis. Next, we provided a diagram of metastatic progression (see Figures 2A and 3A). We explained that the diagram was simply a rough draft intended to initiate discussion, and invited comments and edits. We then asked additional questions to elicit observations and speculations about metastasis and related controversies. In the second part of the interview, we asked experts about specific topics derived from the pre-interview pilot study. Particular questions of interest for modeling metastasis are: When do cells acquire metastatic abilities? What is the basis and the importance of tropism? What (if any) is the relationship between metastasis, development and evolution?

### Analysis of the interviews

All interviews were audio-recorded, transcribed and manually annotated and converted into structured statements. These were grouped by semantic similarity and coded by strength of certainty or conviction, then linked to transcripts and expert identifiers.

### Analysis of the non-uniformity of topics

In addition to qualitative analyses, we tested for non-uniformity in the distribution of the ideas discussed by experts. The null hypothesis in this analysis is that comments are distributed randomly and uniformly across all topics. Under the null hypothesis we can compute the expected number of ideas for each topic and test differences between expected values (i.e., proportional attention) and observed values using a chi-squared statistic with one degree of freedom:
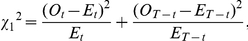
where the subscript *t* refers to the number of comments (ideas) per selected topic, while *T–t* refers to the remaining comments with this particular topic excluded. This calculation implies that the whole collection of comments (ideas) is partitioned into two unequal parts—those focused on *t* and those not (*T-t*). The expected numbers of comments for each part are computed under the hypothesis of a uniform distribution of comments over all topics. To correct for multiple statistical testing for topic-wise analysis, we used a conservative Bonferroni correction requiring a pooled significance of *p*<0.05. We performed a similar analysis of the distribution of comments among expert groups. The goal of this analysis was to identify differences in expert responses across topics and the variation of interest across expert groups.

### Story similarity metrics

**Figure 4:**
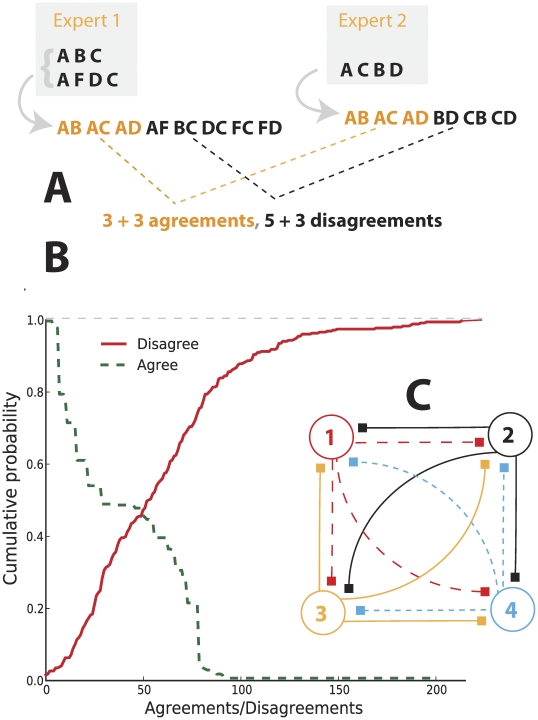
Quantifying the agreement between expert scenarios of metastasis. (A) A hypothetical example of scenarios produced by two experts (experts 1 and 2). Expert 1 suggested two alternative scenarios (ABC and AFDC), while expert 2 suggested only one (ACBD). We define the agreement/similarity between experts as twice the number of stage pairs they share, of all possible ordered pairs of stages from each scenario. We define the disagreement/dissimilarity as the agreement minus the sum of unmatched pairs; see the example. (B) Cumulative probabilities that two experts agree at least at level *x* (similarity of *x* or greater), and that two experts disagree at level *x* or less. The probability of agreement drops rapidly as the number of statements from each expert increases, while the probability of disagreement grows gradually to a rather large number of pairwise disputes. (C) A hypothetical four-expert “regulatory deadlock” could occur if any one of the experts insisted on his disagreement with all others.

To quantify similarity and dissimilarity between pairs of experts, we introduced a metric that compares two series of elements (see Figure 4A). Imagine that two experts' stories included events A, B, C, D, and F, where each letter corresponds to one of the 26 metastasis stages that we or our informants proposed (see Figure 3). One hypothetical expert (Expert 1 in Figure 4A) provided two alternative stories, ABC and AFDC, and the other (Expert 2) only one, ACBD. We first reduce each series to a set of ordered pairs and then combine multiple series from the same expert into a set of unique ordered pairs. These pairs include elements that abut each other in the sequence (AB, BC, CD, etc.) as well as all noncontiguous ordered elements (AC, AD, BD, etc.). Then, we can define similarity (*S_ij_*) between two experts' views (experts *i* and *j*) as twice the number of ordered pairs common to the two sets (*S_ij_ = S_ii_+S_jj_*), and dissimilarity (*D_ij_*) as similarity minus the sum of all unmatched pairs of events from the two sets (see Figure 4A):
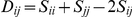
One could adjust *S_ij_* and *D_ij_* by weighting elements (and their associated pairs) by claimed importance, but we neither sought nor obtained unambiguous indications of import in the current interviews.

### Modeling agreement

In order to estimate the nature of expert agreement about the sequence of events in metastasis [15], [16], and also create a standard against which experimental and observational data about metastasis could be evaluated, we built a model that incorporates all expert scenarios as special cases. Our model builds on the simple yet powerful Markov chain formalism [17], [18], which is particularly appropriate given the sequential nature of metastasis. A Markov chain is an ordered sequence of random variables *X*_0_, *X*_1_, …, *X_K_*, which correspond to the sequence of possible metastatic events. Each of these variables can take values (*X*_0_ = *x*_0_, *X*_1_ = *x*_1_, …, *X*_K_ = *x*_K_) from a set of states Σ = {1, 2, …, *N*} that correspond to the expert proposed stages of metastasis (e.g., primary tumor, detachment, invasion, etc. such that *N* = 28—see labels associated with the matrix we discuss in “Integrating expert stories” under “Results”).

The sequence of random variables follows a homogeneous Markov process when
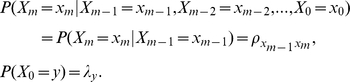
This implies that each metastatic stage depends only on the stage immediately prior, and that the transition probabilities from one stage to another do not vary across the sequence. In this way, the Markov process is determined by a vector and a matrix
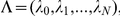
and
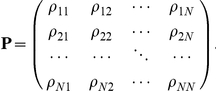
where *ρ_ij_* is the conditional probability of transition from stage *i* to stage *j* so that
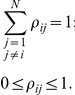
(Note that in general, diagonal elements in a stochastic matrix defining a Markov process can be positive. Because this analysis focuses on the *sequence* of metastatic stages rather than the timing involved in the transition between stages, we do not allow a metastatic stage to transition to itself.)
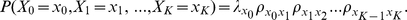
We define *N* states of the process, and assume that it always starts at artificially defined stage S (state 0), and always ends at another artificially defined stage E (state *N*, where *N* = 28). In other words, we start in state S with probability 1 such that
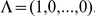
and we always end the chain once state E is reached such that the steady state or stationary distribution vector of metastatic stages (*π*) is defined as
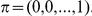
For data with m_i_ observed transitions from state i to any other state, the set of observed counts of transitions across all expert stories, {*c_ij_*}, *_j_*_ = 1,…,*N*_ (where *i≠j*) follows a multinomial distribution with expected values {*ρ_ij_*}, *_j_*_ = 1,…,*N*_

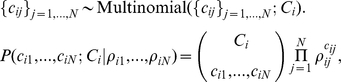
where
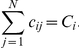
The prior distribution of transition probabilities can then be defined by a Dirichlet distribution
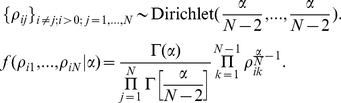
As is customary in these applications [19], we assume that *α*≥*N*-2 (e.g., *α* = 1.1 *N*). The posterior distribution for *ρ_ij_* then is also a conjugate Dirichlet distribution
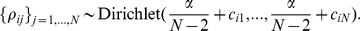
The posterior expectation estimate of *ρ_ij_* is given by
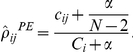
We also obtain a maximum a posteriori probability estimate of *ρ_ij_*:
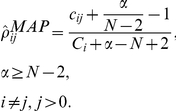



### Certification

The study was approved by the University of Chicago Institutional Review Board.

## Results

### The interviews

We performed, digitally recorded, and transcribed into text 28 interviews with faculty-level experts in metastasis. The duration of these interviews ranged from 15 minutes and 1,579 words to 93 minutes and 11,702 words, with a 39-minute, 4,497 word average. The total duration of our recorded interviews is 18 hours and 3 minutes or 125,916 words.

We grouped our experts in several ways, by the following traits. First, we distinguished them by training background and certification: 15 of our experts held only a PhD; 7 held only an MD; and 6 were MD/PhDs. Second, we divided them by gender, into groups of 19 males and 9 females. Third, we clustered our experts by the period in which they received their first advanced degree: before 1986 (9), between 1986 and 1995 (11), or after 1995 (8). Finally, to control for the context of our interviews, we aggregated those conducted in person with experts from the University of Chicago (16) versus those done over the telephone with experts elsewhere (12).

### Importance of steps in metastasis progression; ordering steps

During our interviews we asked all 28 experts to comment on “our” scheme of metastasis progression. Figure 2A depicts a slightly scrambled schematic of metastasis that we showed to experts as the baseline, expecting corrections and rearrangements. Experts described their understanding of the process, suggesting additions, deletions, and event-ordering changes to the diagram. The overall importance of different stages according to these experts is illustrated in Figure 2B, C, D, and E. While they generally accepted individual stages of the canonical model, experts suggested additional details, rearrangements and clarifications. Biologists (PhDs, see Figure 2B) were more oriented towards the definition of fine-scale molecular events, such as extracellular matrix (ECM) remodeling, adhesion of cancer cells, and individual growth stages of the secondary tumor than were MDs or MD/PhDs (Figures 2C and D). Both PhDs and MDs referred to Paget's “seed and soil” hypothesis when discussing the seeding of cancer cells and finding a niche (“soil”) for a new tumor (Figures 2B and C). As expected, medically trained experts were more inclined to comment on clinical issues, while biologists leaned towards molecular events and mechanisms. In general, all experts agreed on the important events in metastasis, but not on their order.

### Expert-specific stories

The main discrepancy between reviewers was neither their descriptions of individual steps nor their relative importance, but in how they ordered those steps and the granularity of their description of metastasis (Figure 3). Comparing “stories” provided by the 28 experts with each other, we were surprised to find that no two were identical. In their description of metastasis, experts grouped the same symbols/events differently, they varied their ordering of events, and often suggested recurrent events absent in the outline that we showed them. While some disagreements were minor, such as proposing that “some unknown extra steps occur between these events,” others were substantial (see Figure 3A, B, and C). Compare, for example, the “stories” provided by experts 14 and 26.

It was clear after 28 interviews that despite similarities, experts think differently about metastasis. To quantify similarity and dissimilarity between pairs of experts, we introduced a metric that compares two series of elements (Figure 4A and “Materials and Methods” section). This measure allows us to compute the probability that a pair of randomly chosen experts agree on at least *k* statements (*k* = 0, 1, 2, …), or disagree on fewer than *k* statements (see Figure 4B). The probability of agreement drops rapidly with increasing values of *k*, while the probability of disagreement grows more gradually to a rather large number of pairwise disputes (>120). If these views were held to be exclusive and experts did not allow alternative interpretations, we might observe a situation that could be called “regulatory deadlock,” in which any researcher could disagree with all others (Figure 4C) when reviewing their models of metastasis in a manuscript submission or grant proposal.

### Expert responses to specific questions

As we explained in “Materials and Methods”, in the second part of each interview, we asked experts about specific topics. Here we examine five topics that experts discussed, pertinent to computational modeling: (1) definition of the process, (2) stage at which metastasis is acquired, (3) importance of tropism, and (4) connections that link metastasis with development and evolution. Numbers following quotes in the text below are the number of experts that nonexclusively mentioned the idea in question. Note that these numbers should be interpreted as the salience of the topic for that number of experts, which is less than the number that would likely assent to the topic's importance if presented to them. All topics, grouped by expert, can be found in supplementary Dataset S1.

1. When asked, at the beginning of the interview, the simple question “What is metastasis?” some experts defined it broadly with respect to its disease domain (“metastasis is part of cancer” (5)) and others by its formal structure (“metastasis is a multistage process” (6)). Some admitted, “it is not clear what it is” (3). Most, however, also provided more specific diagnostics: defining it as “the spread [of cancer]” (15) or, alternately “the growth of a secondary tumor” (10). Several mentioned specific “steps in the process” or factors that should be distinguished in an appropriate definition. These included “the difference between spread and secondary tumor” (9), the particular “route” of the process (8), the “distant vs. regional” range of spread (5), and whether and how clinical “detection” is possible (4).

2. Experts were asked at which stage they think a cancer cell acquires metastatic abilities. This is a critical topic for modeling as it can be seen as the start of the metastatic process. In this paper we discuss the two main issues on which experts commented: “when” metastatic abilities are acquired and on what factors that acquisition “depends.” (Some experts also commented on a third category, “which cells”, which we display in the supplementary materials - Dataset S1.)

Many experts thought that cancer cells acquire metastatic abilities “early, at the beginning” (12). Several others suggested that “it varies” (10), or specifically that the acquisition took place “throughout” (4) or “during or late in the tumor development” (2). Several suggested the more nuanced view that “the ability is pre-present but changes [are] needed” (6). These could include non-cancer mutations, or forces of stress and pressure on the cell. Others concurred that metastatic abilities were acquired “after mutations” (4), or either “early or after mutations” (3). A single expert mentioned the dependence of metastatic ability on “selective pressure” and many simply stated that they did not know (10). Six transposed the question to *where* the ability is acquired by locating it within “cancer stem cells” (6).

More experts spontaneously noted that acquiring metastatic ability has something to do with an “individual's genetics” (6) than with their “environment” (2). Most thought that acquiring the ability depends on anatomical characteristics ranging from “cell type” (6), “organ site” (1), “tissue stroma and microenvironment” (6) to proximity to “blood supply” (3) or the “extracellular matrix” (2) and the amount of local “biochemical signaling” (3). Others viewed acquisition with regard to dynamic forces like “selection pressure and microenvironment” (6) and “mutations and changes” (8). Many noted acquisition of these abilities hinged on the nature of the tumor (“tumor type, size or stage” (9)). A few also described acquisition functionally—in terms of “loss of suppression” (2), whether the cancer can “successfully evade the immune system” (2), its “ability to move and invade” (3) and to “pass thresholds and barriers” (1). Many admitted nonspecifically that “many factors throughout” (8) were involved, fewer that “it is random” (4), and three confessed ignorance (3).

3. It is known that given the location of a primary tumor, cancer tends to metastasize selectively into different body parts. This phenomenon is termed tropism. Understanding how this selection takes place is critical for modeling metastasis.

We asked experts about the basis for metastasis' tropism and the importance of the relationship between the metastatic cells and their tumor of origin. Experts overwhelmingly associated the basis of tropism with the “microenvironment” (17) of the secondary tumor. Some more specifically detailed that it was involved with “circulation” (10) and “blood supply” (2). Some described the site selection as a nonspecific “stickiness” (3) between primary and secondary tumor sites, and others that it resulted from a process of “selection” (2).

Experts also discussed factors that involve the relationship between primary and metastatic sites. Several mentioned the relevance of the theory of “seed and soil” (7). Some specifically located this relation in the “similar biology in primary and metastatic sites” (7). Others suggested that the relation between seed and soil implied a “developmental connection” (3), that the sites shared “genetic” factors (4) or were linked via “chemical communication” (4). Many experts felt unsure of the relationship, citing “other factors” (6), a nonspecific “combination of things” (9), and that they simply did not know (10).

4. For many years there have been comments in the literature [15], [16] that the metastatic process resembles other processes such as those of (embryonic) development and evolution. In computational modeling, these resemblances could be formalized and exploited.

The relationship between metastasis and (embryonic) development was the topic that produced the most closely related groups of ideas discussed by experts. Most experts suggested that we “need to understand the connection between development and metastasis” (12) or “cancer” in general (3). Nearly everyone noted “similarities between development and cancer and metastasis” (22) (15 separately noted similarities between “development and metastasis” and 14 between “development and cancer”). More specifically, several experts discussed that “cells use existing mechanisms” (9) in the metastasis process. At the same time, however, many noted key “differences between development and cancer and metastasis” (12). Several noted that “development is controlled whereas cancer is not” (10) and some of these and others considered “specific issues on control loss” (10). A few also claimed that “genetic changes” separate the two processes (3).

Most experts made “general comments” (18) of agreement regarding the connection between the processes of metastasis and evolution and many extensively discussed the apparent homology between “natural selection” (17) and metastasis. A few experts also noted that the process of metastatic “treatment is similar to evolution” (3) in the way that cancers come to evade it. Some experts explored “other evolutionary connections” (6).

### What were experts certain about?

**Figure 5:**
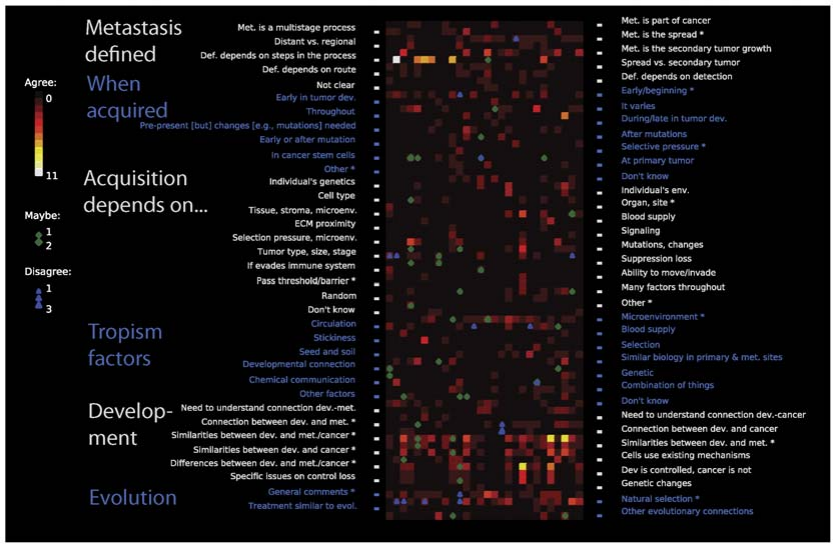
Agreement across experts by discussion topics. We grouped the comments of experts into several topics. Within each topic (represented here by a row), an expert may have mentioned several ideas he or she agrees with, disagrees with, or is unsure about. This heat map depicts the main topics for modeling the process and how many ideas under each topic every expert (columns: 1–28) discussed, along with their agreement. An asterisk (*) indicates a topic whose frequency of mention was statistically significant compared to the null model of a uniform distribution of comments (see Table S1). Abbreviations: *Def* = Definition, *Dev* = Development, *Env* = Environment, *Evol* = Evolution, *Met* = Metastasis, *Mic* = Micro, *Trop* = Tropism.

In total, we recorded 776 comments (27.7 per expert on average), see Figure 5. The majority (715) framed comments about the proposed topics in terms of positive knowledge claims (“agree”), 43 expressed uncertainty (“maybe”), and only 18 mentioned ideas with which they disagreed (“disagree”). On average, men generated slightly more comments than women (28 vs. 26 comments). Most of the positive comments (224) were about the connection between metastasis and development. Specific examples include: “Cancer stem cells are very different from embryonic cells,” and “Embryonic cells are more related to adult normal stem cells.” The least mentioned topic (70 comments) with the most disagreement (5) concerned the connection between metastasis and evolution. Categories that elicited the most uncertainty were metastasis tropism factors (13 “maybe” comments), and on what acquiring metastasis depends (12 “maybes”). See supplementary Dataset S1 for more detailed information.

### Experts' interests/responses in light of their backgrounds

**Figure 6:**
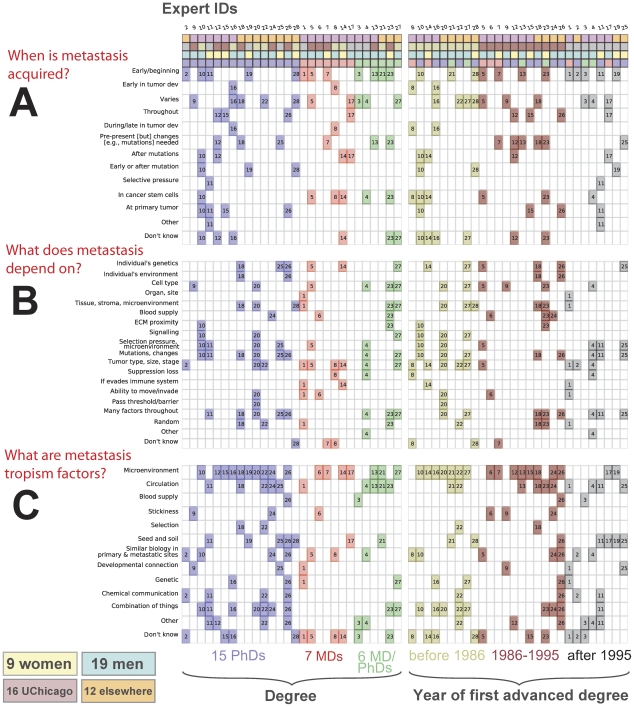
A map of comments provided by experts with details on expert backgrounds. The ideas discussed by the experts for three main interview topics were (A) what acquisition of metastasis requires, (B) when metastasis is acquired, and (C) what factors affect preferential choice of specific tissues/organs by metastatic cells (*tropism*). Color-filled squares indicate that the corresponding idea (one per row) was discussed by the corresponding expert (one per column). The responses have been grouped to illustrate the variance for “PhDs *vs.* MDs *vs.* MD/PhDs” and experts that received their first aforementioned degree “before 1986 *vs.* between 1986 and 1995 *vs.* after 1995.” On the top, the expert ID number can be found followed by four rows of color-coded squares, indicative of four classifications associated with each expert: (1) “The-University-of-Chicago *vs.* elsewhere”; (2) when experts received their first professional degree—“before 1986 *vs.* between 1986 and 1995 *vs.* after 1995”; (3) “men *vs.* women”; (4) and the nature of their advanced degrees—“PhDs *vs.* MDs *vs.* MD/PhDs.”

Expert backgrounds, such as medical or biological training, could influence views and convictions. To detect such trends, we analyzed experts' comments in the context of their backgrounds, classifying experts by training, gender, time of training, and institutional affiliation (Figure 6). These groups significantly differ in their response to some specific issues, calculated with a *χ* ^2^-test. For example, women from our sample were more likely to define metastasis as relating to secondary tumor growth than men (*p*<.01; see supplementary materials for other specific differences). Due to our small sample size, however, differences between groups on the aggregated topics we report below were not statistically significant and should be considered as merely suggestive. We summarize these differences in terms of the percentage of responses that would have occurred if every person had discussed every issue mentioned by anyone. This calculation corresponds to the percentage of “colored” squares from the matrix in Figure 6. Across all groups, lower percentages suggest more diversity of opinion. Differences between groups suggest that some groups consider more issues than others.

When the definition of metastasis was raised, our respondents discussed 72 out of 280 “possible” responses, or 26%. Those who received their first advanced degree between 1986 and 1995 responded with the most issues regarding the definition of metastasis (29% versus 26%), while MDs discussed the fewest (20%). All groups of experts commented approximately equally on the issue of “When is metastasis acquired” (61 out of 364 = 17%). We collected the sparsest or most diverse pattern of response in association with the topic “Acquiring metastasis depends on…” (76 boxes colored out of 532 = 14%). Experts who received their training before 1986 commented on the greatest number of ideas (30/171 or 18%) and those in the 1986–1995 training group commented with the least (25/209 = 12%).

PhDs mentioned the most ideas (27% of the PhD boxes are colored) in discussing metastasis tropism factors (84 boxes colored out of 364 = 23%), while MDs commented with fewest (16%). The densest comments and most statistically significant variation across groups occurred for topics related to “Metastasis and development” (121 boxes colored out of 336 = 36%; see supplementary materials for specific differences). PhDs (42%) and MD/PhDs (43%) commented with the most ideas; and MDs commented with the fewest (25%).

The sparsest comments came from the 1986–1995 training group on the topic “Acquiring metastasis depends on…” (12%), while the most dense commenting was provided by experts educated before 1986 on the topic “Metastasis and development” (42%). Overall, PhDs and MD/PhDs commented with the most ideas (24% and 25%), while MDs commented with fewer (19%). This likely suggests that a clinical focus is less likely to measure, identify or imagine subtle, microscopic stages internal to the process of metastasis.

### Integrating expert stories

If we simply counted ordered pairs of metastatic stages across the entire pool of experts, we could identify those that occur most frequently. These pairs indicate the mode of the agreement distribution. We made an additional effort to create a Markov model to integrate all expert scenarios. Our simple model formalizes the precise distribution of agreement about the progression of metastasis. It also suggests the logical consequences that would result from following the divergent metastatic paths hypothesized by different experts. As such, it creates a standard against which experimental and observational data about metastasis can be evaluated.

**Figure 7:**
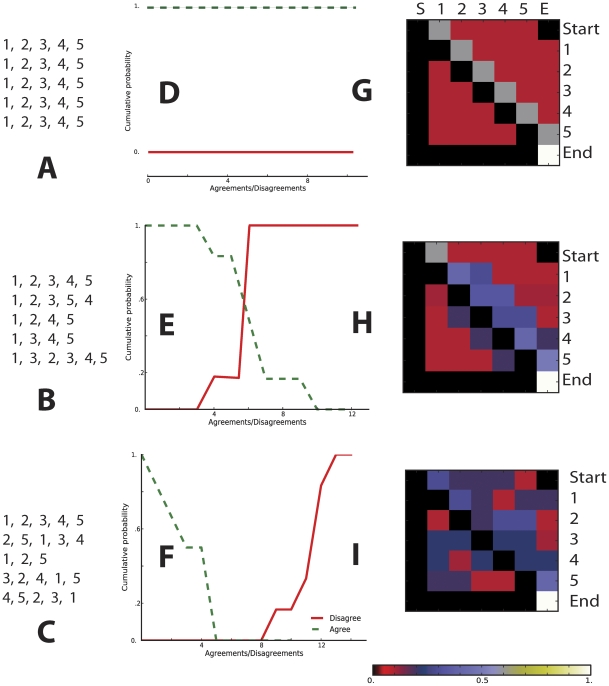
Three hypothetical scenarios involving five experts, each providing a story containing five stages. The three scenarios illustrate situations of (A) “complete agreement”, (B) “moderate agreement”, and (C) “random agreement.” Panels D, E and F illustrate the probability of agreement and disagreement between two randomly chosen experts on at least k statements for each scenario (see Figure 4B). Panels G, H and I render heat maps to illustrate the transition probability matrices after a single Markov chain transition under each scenario. Each stochastic matrix is square, non-negative and organized in the following way. The *i* ^th^ row of the matrix provides probabilities of transitions from state *i* to all other states of the model. The sum of probabilities in each row is therefore equal to 1.

To introduce our model, we first illustrate three, simplified scenarios in Figure 7. In the first (7A), five hypothetical experts provide five stories, each involving five identically ordered stages. In the second (7B), experts provide somewhat different stories and in the third (7C), stories are dramatically different, random re-orderings and deletions of stages. Panels 7D, E and F graph the probability that a pair of randomly chosen experts from each scenario would agree on at least *k* statements (compare with Figure 4B). Panels 7G, H and I visualize transition probabilities in consequence of a single transition for Markov models that integrate all stories from each scenario. For example, 7G illustrates how the Markov model for the “complete agreement” scenario pools virtually all transition probability into the cells just right of the diagonal such that each stage leads almost unequivocally to the next. In contrast, the “random agreement” scenario gives rise to a high-entropy pattern of transition probabilities pictures in 7I. The “moderate agreement” scenario of 7H is a more realistic situation, with most of the transition probability pooling just above and below the diagonal, between stages that most experts believe are near one another.

**Figure 8:**
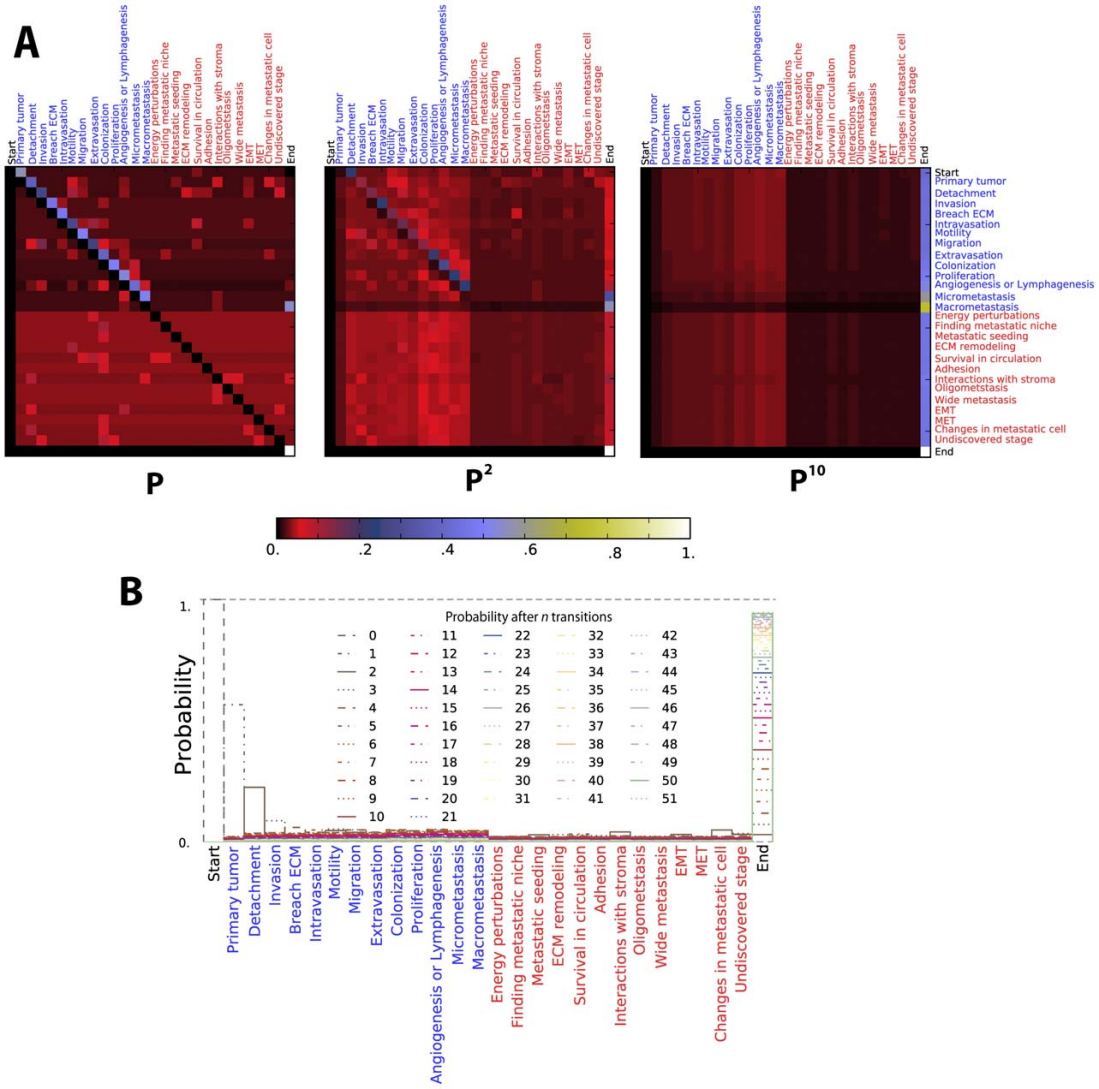
Heat maps that visualize the transition probability matrices in our Markov model of metastasis after *n* Markov chain transitions (*n* = 1, 2, 10); and a plot of probabilities associated with reaching each state after *n* chain transitions. (A) Stochastic matrices are organized as in Figure 7G, H and I: each stochastic matrix is square, non-negative and organized such that the *i* ^th^ row of the matrix provides probabilities of transitions from state *i* to all other states in the model and the sum of each row is equal to 1. The three matrices show the transition probabilities from states *i* to state *j* after one (**P** ^1^), two (**P** ^2^), and ten (**P** ^10^), transition steps of the Markov process. The labels, “Start” and “End” correspond to the generating and absorbing states of the Markov chain, respectively. The rest of the labels indicate metastasis stages that were suggested by the authors (in blue) and by individual experts (in red). (B) The probability of finding the Markov process in a given state at transition *n* (*n* = 1, 2, …, 50) is given by 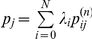. Here **Λ** is the distribution over all states at the beginning of the chain, and **P** *^n^* = [*p_ij_* ^(n)^] is the transition probability matrix after the *n* ^th^ transition (obtained by raising matrix **P** to *n* ^th^ power). This figure demonstrates the decreasing probability that expert intuition about metastasis lands in any particular state at any particular stage. Note that even after 50 transitions there is a substantial probability that the chain would not reach the “End” state.

We visualize the actual transition probabilities between real stages of metastasis for one, two and 10 Markov transition steps in Figure 8 (the data can be found in supplementary Datasets S2, S3, S4, S5, S6). Expert consensus about metastasis corresponds to the most probable path from “start” to “end” state. Specifically, this highest-probability path follows the trajectory: “Start”→primary tumor→detachment→invasion→breach ECM→intravasation→motility→migration→extravasation→colonization→proliferation→angiogenesis→micrometastasis→macrometastasis→“end”.

More interesting, however, are weaker links in the consensus chain. Primary tumor nearly always begins the metastatic sequence (except for those considering “liquid tumors” such as leukemia) and micro- and macrometastasis nearly always end it. Detachment from the primary tumor, however, does not lead to invasion for all experts, but to motility, interactions with the stroma, and other changes in the metastatic cell. Similarly, experts note alternative sequences and supply novel stages at several points in the middle of the metastatic process. This is especially true at entry and exit of cancer from the circulatory system. The Markov chain also suggests that the secondary tumors may spawn secondary, tertiary and higher order metastases (this possibility was mentioned by one of our experts, but we did not encode this knowledge explicitly). The formal model also highlights the possibility that metastasis may terminate at an earlier state. For example, a cell disconnected from the primary tumor may fail to enter the bloodstream or find a suitable niche for establishing a new colony.

The three panels of Figure 8A illustrate the sequence of possible shifts between metastatic stages. As each transition involves the multiplication of prior and current transition probabilities, after 10 transitions, the location of the model is much less certain—and more evenly uncertain—than after 1 or 2 transitions. Not surprisingly, most of the stages suggested by individual experts have much lower transition probabilities because they were not conceptually available to most experts.

The power of our model is not simply to quantify consensus and provide another illustration of expert stories, but to integrate and formalize those stories such that new data can be evaluated against them and a quantitative (probability-based) conclusion can be drawn. To do this, the a posteriori probabilities we present here would become the prior probabilities in a new model, and data from experiment or observation would supply the counts from which new posterior probabilities could be calculated.

Our model can be easily expanded to continuous-time Markov chains if precise time information was attached to metastatic stages in the experimental or observational data to be evaluated. We could use more complicated Markov models, for example, introducing higher-order dependencies between states of the Markov chain, for example, assuming
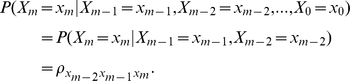
Moreover, the model could be extended to consider partially observed data [20].

Our current model does not account for the fact that some experts collapsed metastatic stages or suggested that they occur in parallel. We could augment the model to accommodate this by creating sub-chains (i.e., start-intravasation, end-intravasation) that include within them all possible sub-stages, and by allowing the introduction of new states to represent simultaneous processes. One might also want to incorporate various expert beliefs about particular issues associated with and affecting certain stages of metastasis (for example, beliefs depicted in Figures 5, 6 and the supplementary files Dataset S1 and Figure S2). Such complexity, however, is beyond the scope of this paper.

## Discussion

A reader may wonder how much time a computational biologist, immersed in designing, streamlining and evaluating mathematical models, should spend learning what biological or clinical experts think. In their search for conceptual clarity, theorists across the sciences feel a natural ambivalence about engaging with the diversity and conflict among perspectives held by experimentalists and observers. We argue passionately, however, that computational biologists, research clinicians, and scientists more broadly should worry about biological and clinical assumptions, probability distributions over expert opinions and subjective prior probabilities associated with conflicting intuitions. If a mathematical model is likened to a building, expert assumptions are the ground beneath. If the underlying soil is swamp, the edifice—the model and its resulting inferences—will sink despite all effort and elegance invested in its design.

In the interest of scientific advance and the computational modeling we believe can accelerate it, we propose that assumptions and hunches be harvested systematically and made electronically available for computational scientists. We believe that if expert knowledge and certainty were referenced as routinely as newly identified nucleotide sequences are queried against established genomes, computational models would be much more scientifically relevant and powerful. Models that take the certainty of assumptions into account will be able to use widely established certainty as an effective modeling constraint. Even more important, computational models will be able to work out the implications and arbitrate between more and less plausible intuitions on the path to greater scientific agreement and better founded certainty. This could be especially significant in emerging areas that concern complex phenomenon like metastasis where much diversity of opinion remains.

How to harvest insights at large scales remains an open problem, but a solvable one. Ideally, many experts in the field should be interviewed. It is rarely feasible to query them all, and so a subset should be sought that maximizes social and educational diversity. This will help to increase the range of independent experiences with the same natural phenomenon. When researchers are part of a close community, they often share assumptions converged upon through communication and social influence. We also believe that emerging methods of harvesting expert assumptions and intuitions show promise, including crowdsourcing and mining text from articles, presentation slides and science blogs.

The results we obtained in this study did not match our expectations. We expected some disagreement, but we did not anticipate the level of conceptual diversity encountered. We anticipated that experts would agree on the majority of issues, while minor matters would generate mild deviation. Instead, we found widely divergent stories with each story distinct from every other. Admittedly, many of these differences were small, as the addition of precise intermediate steps in the metastatic process by molecular and cell biologists. Other differences, however, were large, including whether or not secondary tumors could lead to “tertiary” metastasis. We were also impressed by the positive conviction experts expressed—strong beliefs largely incompatible across scientists and yet perceived and presented as representing the field [20]. This conclusion was partially conditioned on the nature of the field. Metastasis is a complex, multi-faceted phenomenon, investigated by a broad federation of overlapping scientific communities. Nevertheless, many research areas in biomedicine approximate these conditions, including investigations of virtually every complex disorder resulting from a combination of multiple genetic and environmental factors like schizophrenia, coronary heart disease or asthma.

Our analysis also suggests the importance of developing improved methods to analyze competing scientific accounts. In this paper, we only examine pairs of elements, and yet these pairs are part of larger sequences that intersect one another. Previous research that renders narratives as sequences and intersecting networks of claims [21]–[24] suggests exciting possibilities regarding how to identify important subsequences and the centrality of elements in theoretical narratives. We will explore these and other possibilities in future research. Our Markov model provides a first step to formally integrate expert consensus surrounding the stages of metastasis and organize a set of hypotheses against which future experimental and observed data on metastasis can be evaluated. We argue that our method could also enable researchers to gain insight into the diversity of accounts beyond the realms of science. These include historians' competing narratives about a past event, eyewitness reports of a current one, or judicial opinion about a bundle of related legal cases. Markov model formalism can represent not only narrative or logical sequences, but also continuous-space events like the diffusion of an innovation or fad.

Our analysis has broad practical implications for understanding mechanisms through which scientific ideas succeed or fail. Mathematical models will fail if they are based on false biological assumptions. Nevertheless, well-executed grant proposals or manuscripts involving models can also be rejected because they are based on reasonable assumptions not shared by the reviewer. Our analysis of the distribution of expert stories regarding cancer metastasis demonstrates that the probability of disagreement on assumptions is high for any pair of experts (see Figure 4B). Furthermore, when multiple experts review a manuscript, the probability that at least one opposes some of the assumptions in the manuscript approaches unity. Nevertheless, proposals are funded and articles published, implicating a pragmatic social process through which experts certify research with which they disagree. It is unclear, however, what rules guide this process. For example, it may be that research at the boundaries between established perspectives is discounted [25], even though this is the position at which mathematical models and integrative theory could be most useful for the advancement of science.

Our analyses also suggest testable hypotheses about the distribution of expert opinions across the scientific community. We conjecture that in the total population of expert ideas, individual assumptions and hunches can be approximated with a Zipfian or zeta distribution that follows a power law with a heavy tail. In other words, the probability that a particular assumption is shared by *k* experts in the general population of scientists is proportional to *c k^−γ^*, where *γ* is a positive real parameter that is greater or equal to 1 and *c* is a normalizing constant ensuring that the distribution sums to 1 over all allowable *k*'s (1, 2, 3, …). This suggests that a few ideas are widely shared, but that many more are rare, held only by individual scientists or small groups. We base this conjecture on the observation that the generation and propagation of assumptions employs mechanisms similar to those involving the popularity of words in human language, the distribution of which served as the basis for Zipf's law. If this conjecture were true, assumptions from a very large number of experts would be required to approximate the collective knowledge of all experts with fidelity. On the other hand, common assumptions could be captured from analysis of a small number of interviews.

The most critical and vexing issue involves the relationship between the distribution of expert opinion and biological reality. It is unclear whether common ideas result from independent experiences with biological phenomena or represent axioms around which the field has converged as a function of shared training, shared tools, and communication. This key issue merits separate investigation, but regardless of the answer, idiosyncratic knowledge held by scientists in the tail of the distribution cannot be the result of an artificial convergence process. While some of this conceptual idiosyncrasy is invariably the result of logical or experimental error, there is likely much sound, but rare insight in it that, when harvested and incorporated into formal hypotheses like our Markov model, could accelerate the advancement of science.

## Supporting Information

Dataset S1 Excel file with all the data on the topics discussed in this paper.
(XLS)

Dataset S2 Formal representation cascade (text file).
(TXT)

Dataset S3 Formal representation cascade (annotated PDF file).
(PDF)

Dataset S4 Formal representation cascade per expert (text file).
(TXT)

Dataset S5 Formal representation cascade per expert with implicit links (text file).
(TXT)

Dataset S6 Markov matrix estimated in our study.
(TXT)

Figure S1 Fuzzy plots per expert including stage labels – supplement to Figure 3. The plot in Figure S1 also shows variation in naming different parts of the process, whereas “?” denotes uncertainty about the particular part.
(PDF)

Figure S2 Comments for all topics by all expert groups – supplement to Figure 6.
(PDF)

Table S1 Table of statistical significance testing.
(PDF)
