# Supplementary material for: Conflicting Biomedical Assumptions for Mathematical Modeling: The Case of Cancer Metastasis
Source: PLoS Comput Biol. 2011 Oct 6;7(10):e1002132. doi: 10.1371/journal.pcbi.1002132 (PMC3188482; doi:10.1371/journal.pcbi.1002132)
Supplement: Dataset S3 — Formal representation cascade (annotated PDF file). (PDF) [file pcbi.1002132.s003.pdf]

ANNOTATIONS

Node [N]
N1 --> N2 = edge & direction
y = yes (= 100%)
n = no
{} = together
(additional process at) [N]
[ ] = closed boundaries
[N1, N2] = from the beginning of N1 until the end of N2
( ) = open boundaries
(N1, N2) = after the end of N1 and before the beginning of N2
&& = "and", both together
process [N1, N2… Nn] = process at all nodes

	PROPOSED	PhDs (15)	MDs (7)	MD/PhDs (6)	All (28)
Primary [P]	y	12	7	6	25
Detachment [A]	y	11	6.5	6	23.5
Invasion [I]	y	10	5	3	18
Breach ECM [R]	y	9	5	2.5	16.5
Intravasation [V]	y	10.5	4	4	18.5
[B] = {I, R, V}	y	9	4	2	15
Motility [O]	y	9	6	2.5	17.5
Migration [T]	y	9	6	4	19
[C] = {O, T}	y	9	6	2.5	17.5
Extravasation [D]	y	7.5	4	3.5	15
Colonization [L]	y	13	8	6	27
Proliferation [X]	y	13	6	5	24
Angiogenesis [S]	y	12.5	6	4	22.5
[E] = {L, X, S}	y	12	6	4	22
Micrometastasis [F]	y	13	6	6	25
Macrometastasis [G]	y	12	6	5	23
P --> A	y				
A --> B	y				
B --> C	y				
C --> D	y				
D --> E	y				
E --> F	y				
F --> G	y				
{A, B, C}	n	3	2	0	5
A --> C --> B --> E	n	3	1	1	5
C --> {A, B} --> D	n	1	1	0	2
{A, C} --> B --> D	n	1	0	0	1
B --> D --> C --> E	n	0.5	0	0	0.5
(vessel infiltration) [P]	n	0	1	0	1
(interaction with stroma) [P]	n	1	1	0	2
(events at primary) [P]	n	1	0	0	1
(EMT starts) [A]	n	0.5	0	0	0.5
(lymph- & angio- genesis starts) [A]	n	0.5	0	0	0.5
(ECM remodelling) [A]	n	2	0	0	2
(EMT) [B]	n	0.5	0	0	0.5
(lymph- & angio- genesis) [B, D]	n	0.5	0	0	0.5
(lymph- & angio- genesis) [C, E, F, G]	n	1	0	0	1
(progressive growth) [E]	n	2	0	0	2
{E1, E2, E3… En} = [E]	n	1	0	0	1
(interactions with stroma) [F]	n	1	0	0	1
(interactions with stroma) [G]	n	2	0	0	2
(something happens to cell) (P, A)	n	0	0	2	2
go somewhere (P, A)	n	1	0	0	1
survival in circulation (C, D)	n	2	1	1	4
energy pertubations (C, D)	n	1	0	0	1
metastatic niche (D, E)	n	1	1	0	2
trigger (D, E)	n	0	1	0	1
angiogenesis (F, G)	n	0.5	0	1	1.5
oligometastasis [X] && G --> X	n	1	0	0	1
oligometastasis [X] && G --> X && (interactions with stroma) [X]	n	1	0	0	1
oligometastasis [X] && wide metastasis [Z] && G --> X --> Z	n	1	0	0	1
metastatic seeding (A, E)	n	0	1	0	1
EMT starts (P,)	n	1.5	0	0	1.5
floating around (A, C)	n	0	0	1	1
adhesion (A, E)	n	1	0	0	1
angiogenesis (, B)	n	1	0	0	1
MET (, E)	n	1	0	0	1

These were not explicitily commented, therefore no values.

Comments/Explanations

ABC together
C <--> B
C --> AB
AC --> B
D <--> C

0_vessel infiltration
0_interactions with stroma
0_events at primary
A_EMT starts
A_lymph- & angio- genesis starts
A_ECM remodelling
B_EMT
B/D_lymph- & angio- genesis
C/E/F/G_lymph- & angio- genesis
E_(progressive) growth
E --> E1, E2, E3, E4…
F_interactions with stroma
G_interactions with stroma
something happens to cell
go somewhere
survival in circulation
energy pertubations
metastatic niche
trigger
angiogenesis
oligometstasis

interactions with stroma
wide metastasis

metastatic seeding (after A, before E)
EMT starts (after primary)
floating around (after A, before C)
adhesion (after A, before E)
angiogenesis (before B)
MET (before E)
