# Supplementary figures and images for: Conflicting Biomedical Assumptions for Mathematical Modeling: The Case of Cancer Metastasis
Source: PLoS Comput Biol. 2011 Oct 6;7(10):e1002132. doi: 10.1371/journal.pcbi.1002132 (PMC3188482; doi:10.1371/journal.pcbi.1002132)

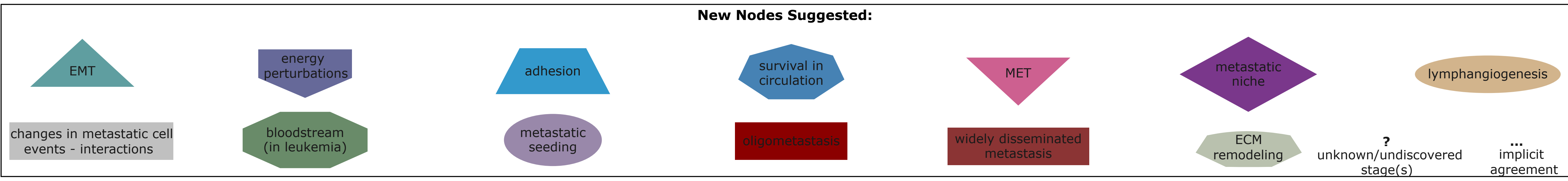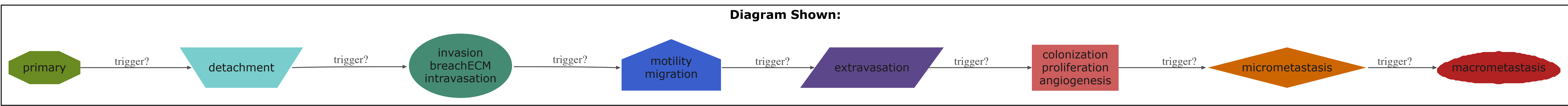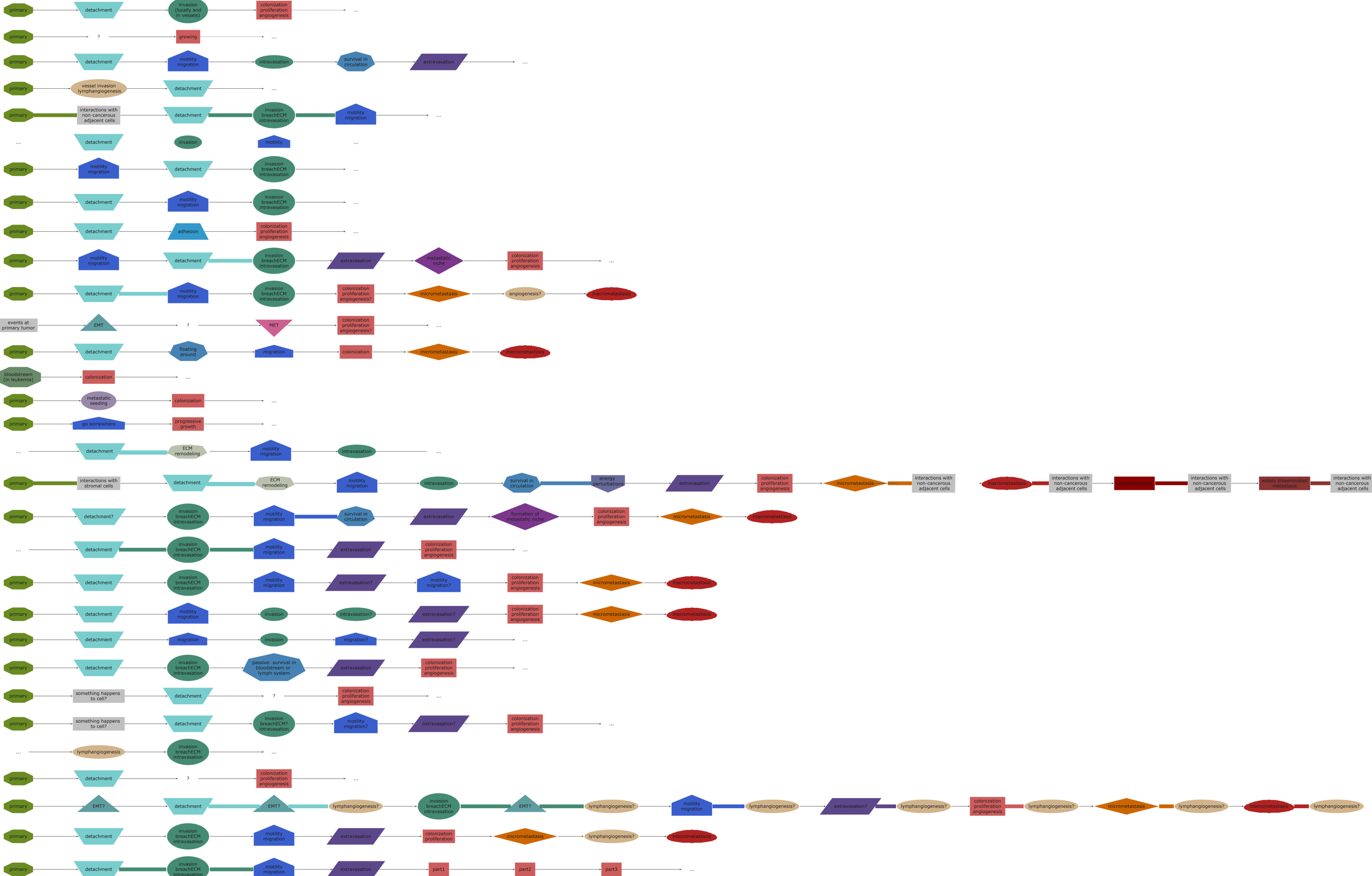

Supplement: Figure S1 — Fuzzy plots per expert including stage labels – supplement to Figure 3. The plot in Figure S1 also shows variation in naming different parts of the process, whereas “?” denotes uncertainty about the particular part. (PDF) [file pcbi.1002132.s007.pdf]
