# Supplementary material for: Conflicting Biomedical Assumptions for Mathematical Modeling: The Case of Cancer Metastasis
Source: PLoS Comput Biol. 2011 Oct 6;7(10):e1002132. doi: 10.1371/journal.pcbi.1002132 (PMC3188482; doi:10.1371/journal.pcbi.1002132)
Supplement: Figure S2 — Comments for all topics by all expert groups – supplement to Figure 6. (PDF) [file pcbi.1002132.s008.pdf]

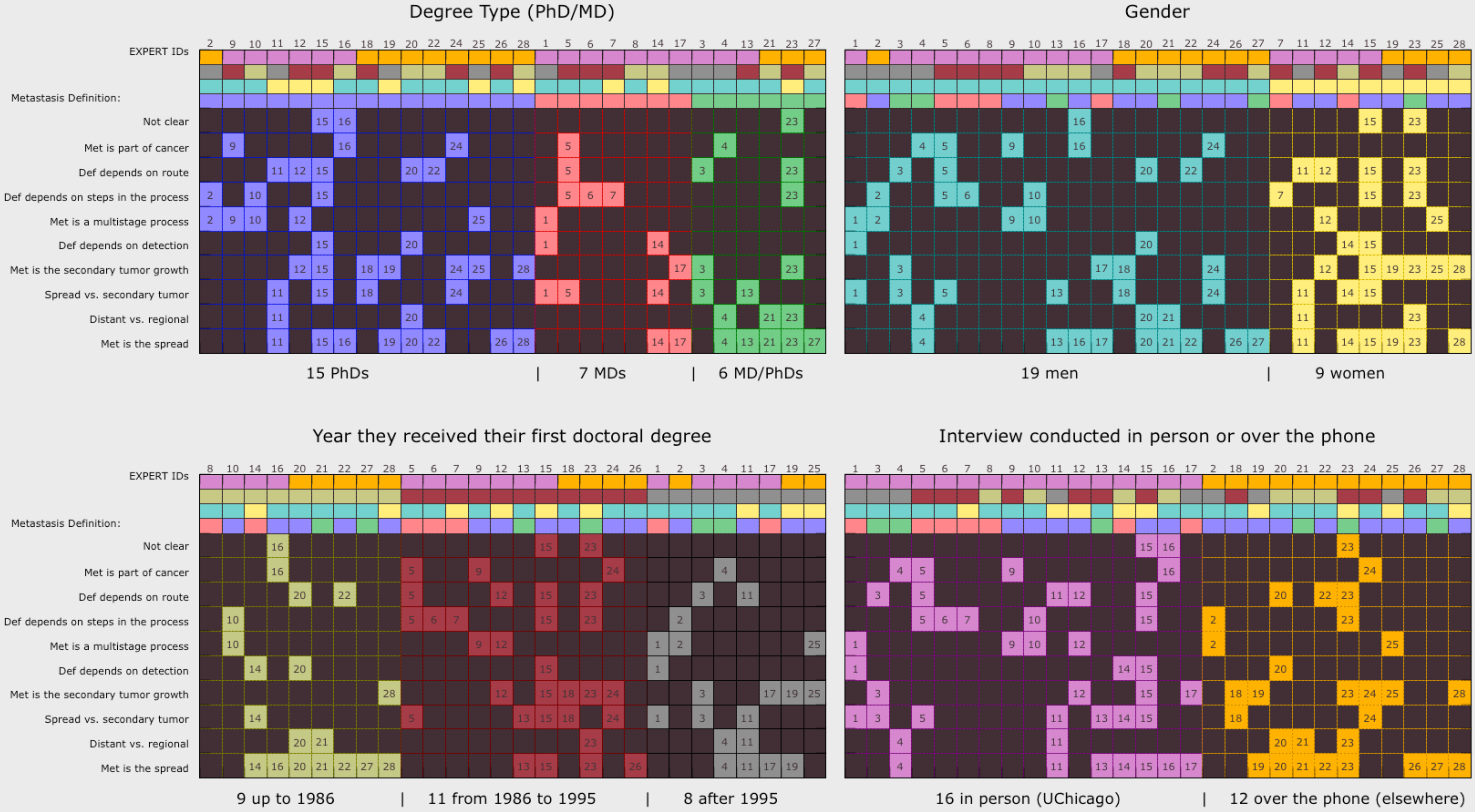

Degree Type (PhD/MD)
EXPERT IDs
2 9 10 11 12 15 16 18 19 20 22 24 25 26 28 1 5 6 7 8 14 17 3 4 13 21 23 27
Metastasis Definition:
Not clear
Met is part of cancer
Def depends on route
Def depends on steps in the process
Met is a multistage process
Def depends on detection
Met is the secondary tumor growth
Spread vs. secondary tumor
Distant vs. regional
Met is the spread
15 PhDs | 7 MDs | 6 MD/PhDs
Gender
1 2 3 4 5 6 8 9 10 13 16 17 18 20 21 22 24 26 27 7 11 12 14 15 19 23 25 28
19 men | 9 women
Year they received their first doctoral degree
EXPERT IDs
8 10 14 16 20 21 22 27 28 5 6 7 9 12 13 15 18 23 24 26 1 2 3 4 11 17 19 25
Metastasis Definition:
Not clear
Met is part of cancer
Def depends on route
Def depends on steps in the process
Met is a multistage process
Def depends on detection
Met is the secondary tumor growth
Spread vs. secondary tumor
Distant vs. regional
Met is the spread
9 up to 1986 | 11 from 1986 to 1995 | 8 after 1995
Interview conducted in person or over the phone
1 3 4 5 6 7 8 9 10 11 12 13 14 15 16 17 2 18 19 20 21 22 23 24 25 26 27 28
16 in person (UChicago) | 12 over the phone (elsewhere)

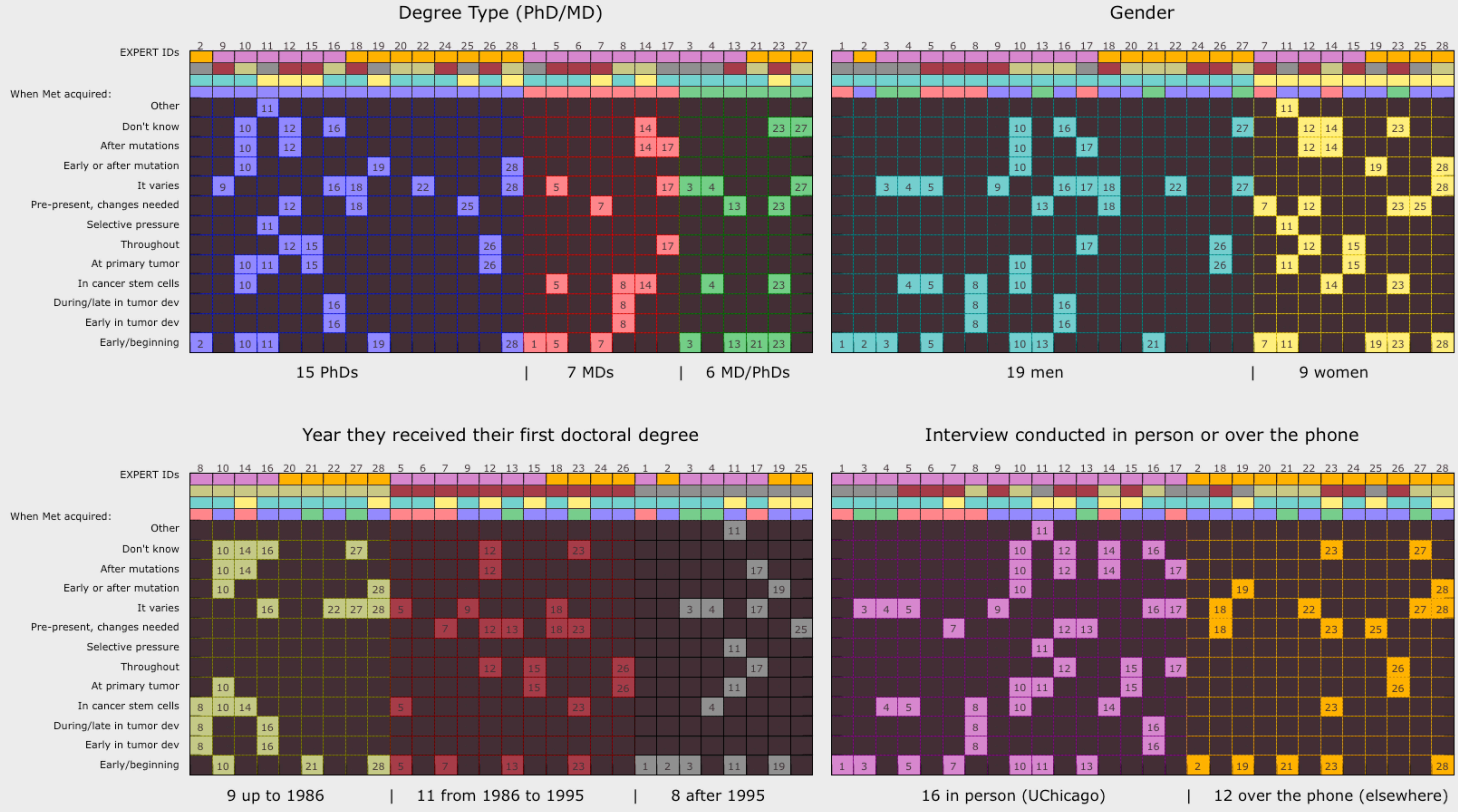
Degree Type (PhD/MD)
Gender
EXPERT IDs
When Met acquired:
Other
Don't know
After mutations
Early or after mutation
It varies
Pre-present, changes needed
Selective pressure
Throughout
At primary tumor
In cancer stem cells
During/late in tumor dev
Early in tumor dev
Early/beginning
15 PhDs | 7 MDs | 6 MD/PhDs
19 men | 9 women
Year they received their first doctoral degree
Interview conducted in person or over the phone
9 up to 1986 | 11 from 1986 to 1995 | 8 after 1995
16 in person (UChicago) | 12 over the phone (elsewhere)

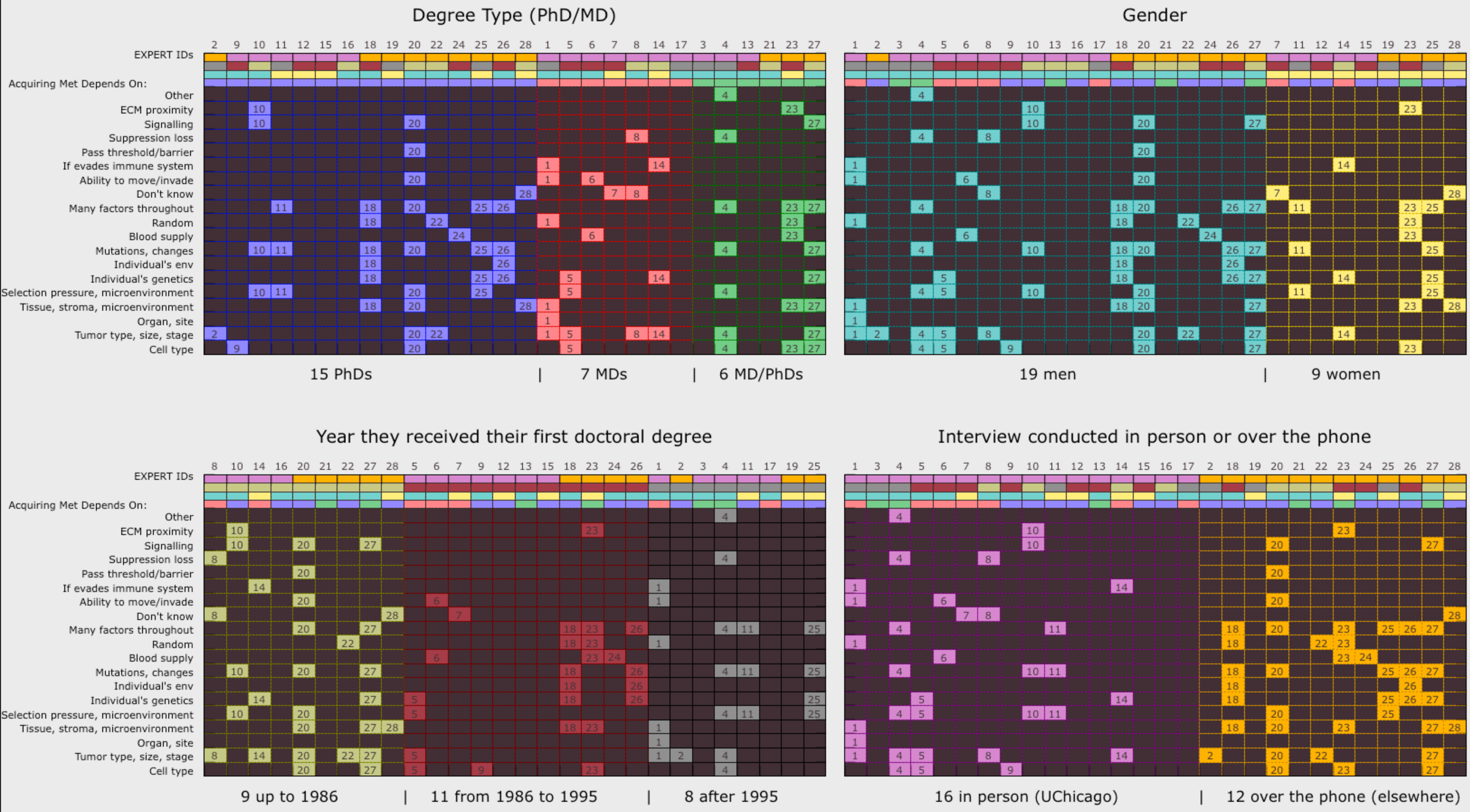

Degree Type (PhD/MD)
Gender
EXPERT IDs
Acquiring Met Depends On:
Other
ECM proximity
Signalling
Suppression loss
Pass threshold/barrier
If evades immune system
Ability to move/invade
Don't know
Many factors throughout
Random
Blood supply
Mutations, changes
Individual's env
Individual's genetics
Selection pressure, microenvironment
Tissue, stroma, microenvironment
Organ, site
Tumor type, size, stage
Cell type
15 PhDs | 7 MDs | 6 MD/PhDs
19 men | 9 women
Year they received their first doctoral degree
Interview conducted in person or over the phone
9 up to 1986 | 11 from 1986 to 1995 | 8 after 1995
16 in person (UChicago) | 12 over the phone (elsewhere)

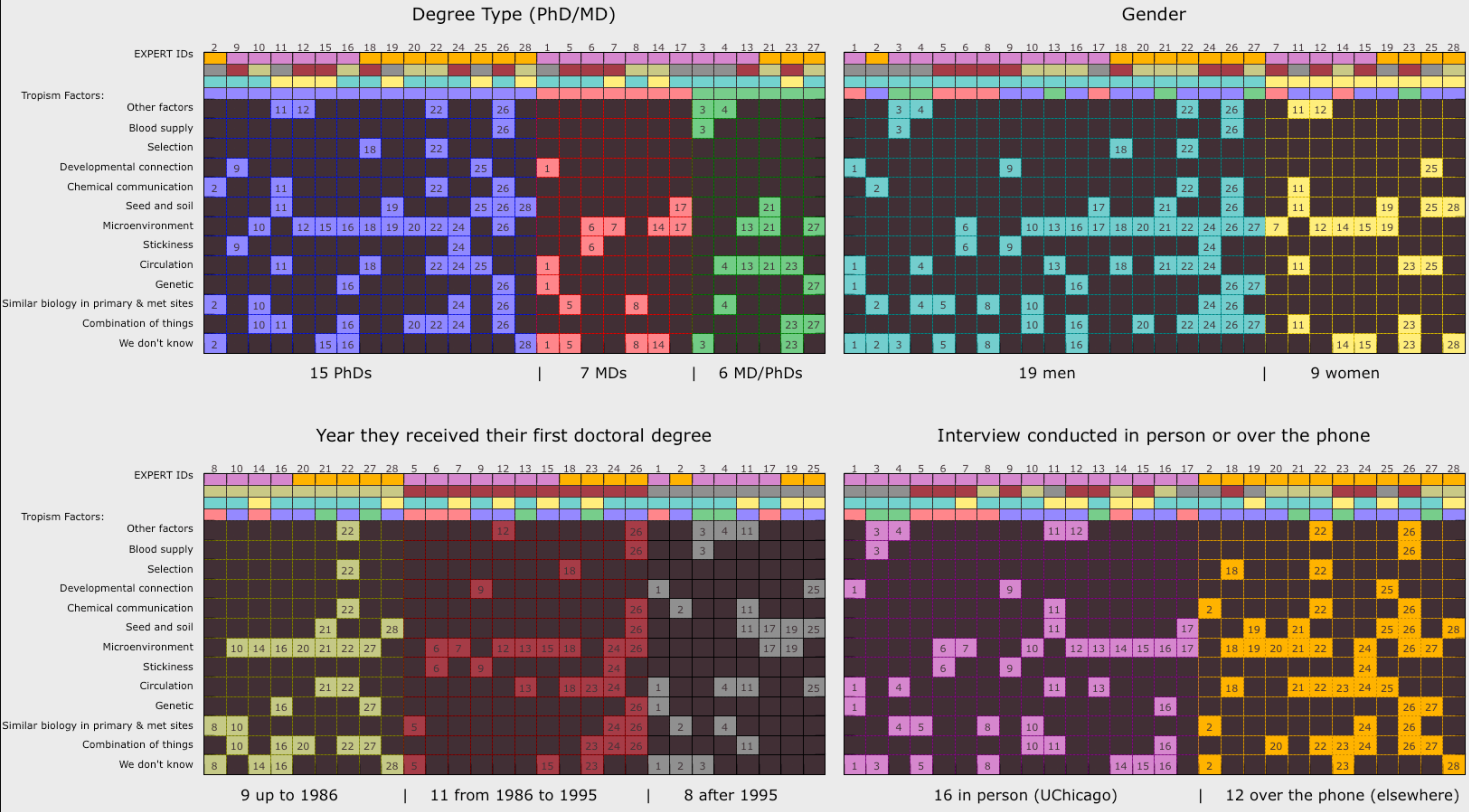

Degree Type (PhD/MD)
Gender
Year they received their first doctoral degree
Interview conducted in person or over the phone
EXPERT IDs
Tropism Factors:
Other factors
Blood supply
Selection
Developmental connection
Chemical communication
Seed and soil
Microenvironment
Stickiness
Circulation
Genetic
Similar biology in primary & met sites
Combination of things
We don't know
15 PhDs | 7 MDs | 6 MD/PhDs
19 men | 9 women
9 up to 1986 | 11 from 1986 to 1995 | 8 after 1995
16 in person (UChicago) | 12 over the phone (elsewhere)

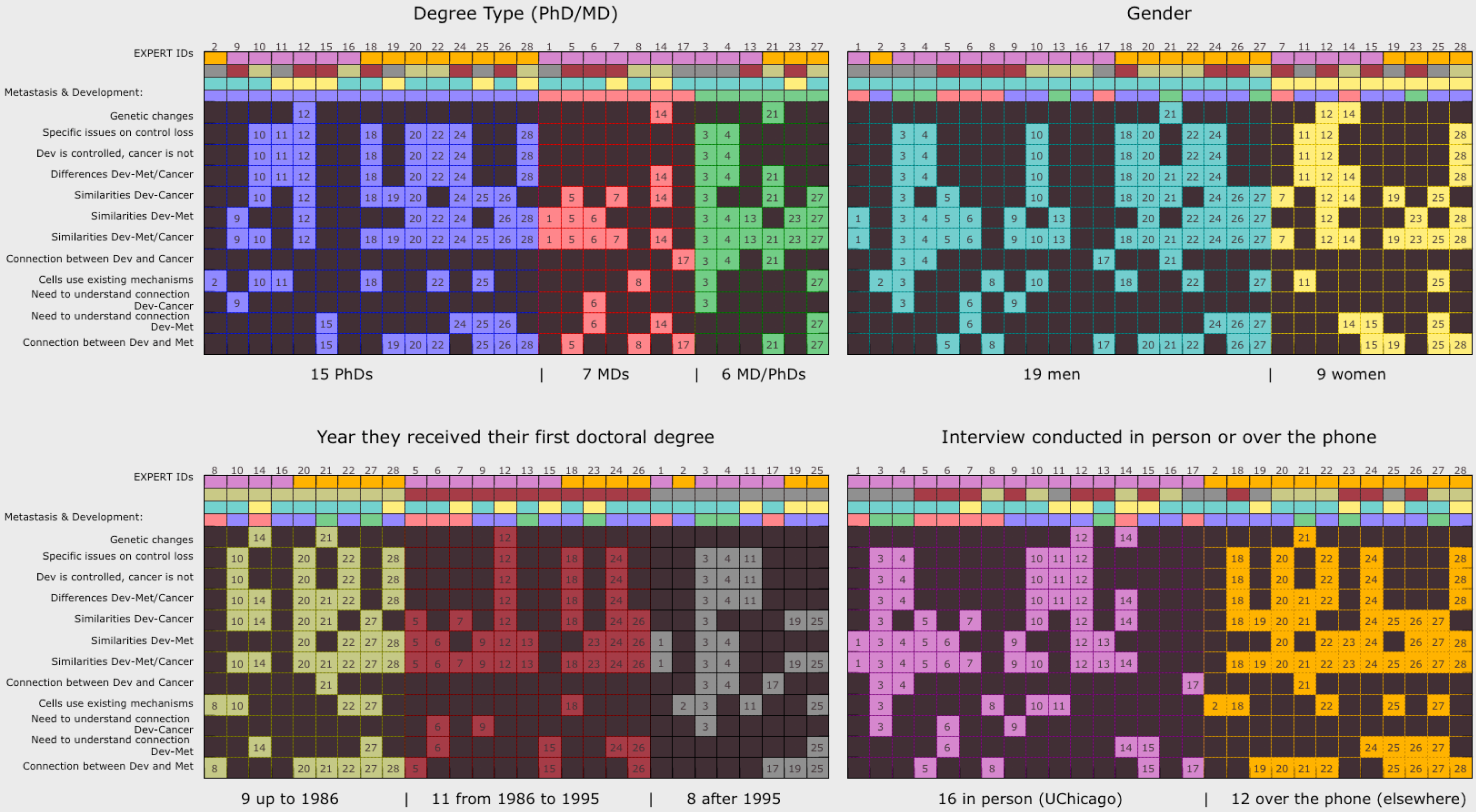
Degree Type (PhD/MD)
Gender
EXPERT IDs
Metastasis & Development:
Genetic changes
Specific issues on control loss
Dev is controlled, cancer is not
Differences Dev-Met/Cancer
Similarities Dev-Cancer
Similarities Dev-Met
Similarities Dev-Met/Cancer
Connection between Dev and Cancer
Cells use existing mechanisms
Need to understand connection Dev-Cancer
Need to understand connection Dev-Met
Connection between Dev and Met
15 PhDs | 7 MDs | 6 MD/PhDs
19 men | 9 women
Year they received their first doctoral degree
Interview conducted in person or over the phone
9 up to 1986 | 11 from 1986 to 1995 | 8 after 1995
16 in person (UChicago) | 12 over the phone (elsewhere)

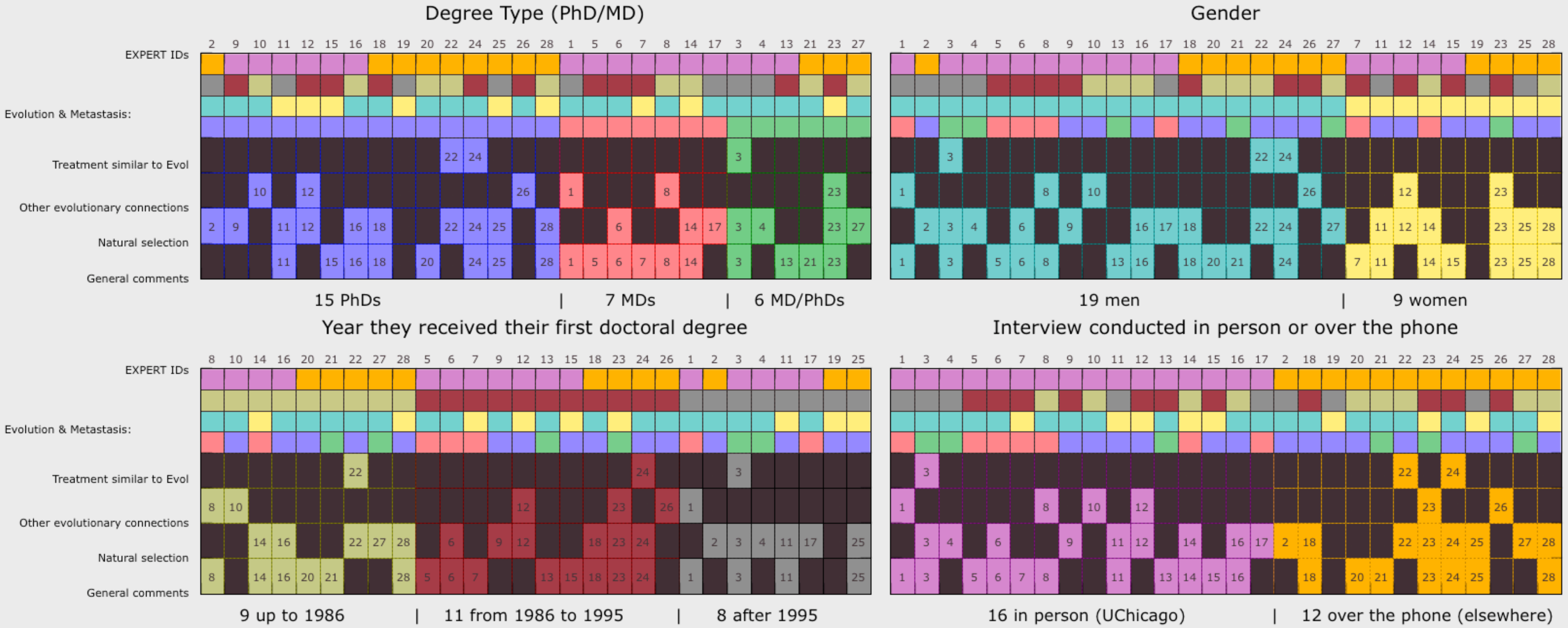
Degree Type (PhD/MD)
2 9 10 11 12 15 16 18 19 20 22 24 25 26 28 1 5 6 7 8 14 17 3 4 13 21 23 27
EXPERT IDs
Evolution & Metastasis:
Treatment similar to Evol
Other evolutionary connections
Natural selection
General comments
15 PhDs | 7 MDs | 6 MD/PhDs
Gender
1 2 3 4 5 6 8 9 10 13 16 17 18 20 21 22 24 26 27 7 11 12 14 15 19 23 25 28
19 men | 9 women
Year they received their first doctoral degree
8 10 14 16 20 21 22 27 28 5 6 7 9 12 13 15 18 23 24 26 1 2 3 4 11 17 19 25
EXPERT IDs
Evolution & Metastasis:
Treatment similar to Evol
Other evolutionary connections
Natural selection
General comments
9 up to 1986 | 11 from 1986 to 1995 | 8 after 1995
Interview conducted in person or over the phone
1 3 4 5 6 7 8 9 10 11 12 13 14 15 16 17 2 18 19 20 21 22 23 24 25 26 27 28
16 in person (UChicago) | 12 over the phone (elsewhere)
