# Supplementary material for: Conflicting Biomedical Assumptions for Mathematical Modeling: The Case of Cancer Metastasis
Source: PLoS Comput Biol. 2011 Oct 6;7(10):e1002132. doi: 10.1371/journal.pcbi.1002132 (PMC3188482; doi:10.1371/journal.pcbi.1002132)
Supplement: Table S1 — Table of statistical significance testing. (PDF) [file pcbi.1002132.s009.pdf]

Topic		Significant topic?	PhDs	MDs	MD/ PhDs	men	women	<86	86-95	>95	not UChicago	UChicago
Definition	Metastasis is the Spread	0.00070	0.01414		0.00206	0.02641	0.00627	0.00061			0.00159	
	Distant vs. Regional											
	Spread vs. secondary tumor											
	Metastasis is the secondary tumor growth						0.00627					
	Definition depends on Detection											
	Metastasis in a Multistage Process											
	Definition depends on Steps in the Process											
	Definition depends on Route											
	Metastasis is part of Cancer											
	Not clear what it is											
Tropism factors	We don't know											
	Combination of things							0.04165				
	Similar biology in primary & metastatic sites											
	Genetic											
	Circulation				0.02576							
	Stickiness											
	Microenvironment	0.00003	0.00041			0.00025	0.04165	0.00061	0.00058		0.00159	0.00545
	Seed and soil											
	Chemical communication											
	Developmental connection											
	Selection											
	Blood supply											
	Other factors											
When Metastasis acquired	Early/beginning	0.02777			0.02576		0.04165			0.01980		
	Early in tumor development											
	During/late in tumor development											
	In cancer stem cells											
	At primary tumor											
	Throughout											
	Selective pressure	0.03067				0.03553						
	Pre-present, changes needed											
	It varies					0.02641						
	Early or after mutation											
	After mutations											
	We don't know											
	Other	0.03067				0.03553						
Acquiring Metastasis Depends On	Cell type											
	Tumor type, size, stage							0.04165				
	Organ, site	0.03067										
	Tissue, stroma, microenvironment											
	Selection pressure, microenvironment											
	Individual's genetics											
	Individual's environment											
	Mutations, changes											
	Blood supply											
	Random											
	Many factors throughout											
	Don't know											
	Ability to move/invade											
	If evades immune system											
	Pass threshold/barrier	0.03067										
	Suppression loss											
	Signaling											
	ECM proximity											
	Other	0.03067										
Developmental Connections	Connection between Development and Metastasis	0.02777						0.00627			0.00159	
	Need to understand connection between Development and Metastasis											
	Need to understand connection between Development and Cancer											
	Cells use existing mechanisms											
	Connection between Development and Cancer											
	Similarities between Development and Metastasis/Cancer	0.00000	0.00005	0.00754	0.00008	0.00000	0.00061	0.00061	0.00000	0.01980	0.00000	0.00013
	Similarities between Development and Metastasis	0.00070			0.00206	0.00025			0.00058		0.01062	0.02407
	Similarities between Development and Cancer	0.00332	0.01414			0.02641	0.04165	0.04165	0.02902		0.00159	
	Differences between Development and Metastasis/Cancer	0.03165	0.01414					0.00627				
	Development is controlled, cancer is not		0.01414									
	Specific issues on control loss		0.01414									
	Genetic changes											
Evolutionary Connections	General comments in agreement	0.00000	0.01414	0.00054	0.02576	0.00147	0.00061	0.00627	0.00058		0.01062	0.00013
	Natural selection	0.00003	0.00041		0.02576	0.00147	0.00627	0.04165		0.00215	0.00159	0.00545
	Other evolutionary connections											
	Treatment similar to Evolution											
